# A minimal helical-hairpin motif provides molecular-level insights into misfolding and pharmacological rescue of CFTR

**DOI:** 10.1038/s42003-018-0153-0

**Published:** 2018-09-28

**Authors:** Georg Krainer, Antoine Treff, Andreas Hartmann, Tracy A. Stone, Mathias Schenkel, Sandro Keller, Charles M. Deber, Michael Schlierf

**Affiliations:** 1 B CUBE—Center for Molecular Bioengineering, Technische Universität Dresden, Arnoldstr. 18, 01307 Dresden, Germany; 2 Molecular Biophysics, Technische Universität Kaiserslautern (TUK), Erwin-Schrödinger-Str. 13, 67663 Kaiserslautern, Germany; 3 Division of Molecular Medicine, Research Institute, Hospital for Sick Children, 686 Bay Street, Toronto, ON M5G 0A4 Canada; 4 Department of Biochemistry, University of Toronto, 1 King’s College Circle, Toronto, ON M5S 1A8, Canada

## Abstract

Our meagre understanding of CFTR misfolding and its reversal by small-molecule correctors hampers the development of mechanism-based therapies of cystic fibrosis. Here we exploit a helical-hairpin construct—the simplest proxy of membrane-protein tertiary contacts—containing CFTR’s transmembrane helices 3 and 4 and its corresponding disease phenotypic mutant V232D to gain molecular-level insights into CFTR misfolding and drug rescue by the corrector Lumacaftor. Using a single-molecule FRET approach to study hairpin conformations in lipid bilayers, we find that the wild-type hairpin is well folded, whereas the V232D mutant assumes an open conformation in bilayer thicknesses mimicking the endoplasmic reticulum. Addition of Lumacaftor reverses the aberrant opening of the mutant hairpin to restore a compact state as in the wild type. The observed membrane escape of the V232D hairpin and its reversal by Lumacaftor complement cell-based analyses of the full-length protein, thereby providing in vivo and in vitro correlates of CFTR misfolding and drug-action mechanisms.

## Abstract (web summary)

Georg Krainer and Antoine Treff et al. use a helical-hairpin construct derived from the cystic fibrosis transmembrane conductance regulator (CFTR) to investigate misfolding caused by the disease-linked V232D mutation. Using single-molecule FRET, they show that the V232D hairpin assumes an open conformation in lipid bilayers, which is reversed by the pharmacological corrector Lumacaftor.

## Introduction

Cystic fibrosis is the most common lethal genetic disease in the Western world^1^. It is caused by mutations in the cystic fibrosis transmembrane conductance regulator (CFTR), a membrane channel that regulates anion flow across epithelial cells^2,3^. Most of the >1000 disease-causing CFTR mutations^4^ disrupt its biogenesis or native fold, ultimately leading to a loss of functional protein at the cell surface. Despite considerable progress in understanding the disease on a cellular level, the exact mechanisms by which mutations influence the conformation and trigger misfolding of CFTR remain obscure. Yet a better understanding of CFTR misfolding is highly desirable for the development of novel therapeutics that treat the cause rather than the symptoms of cystic fibrosis. Numerous small molecules that correct CFTR misfolding and restore channel function have been developed; however, our understanding of their molecular mechanisms of action remains, at best, rudimentary^5–7^.

**Fig. 1:**
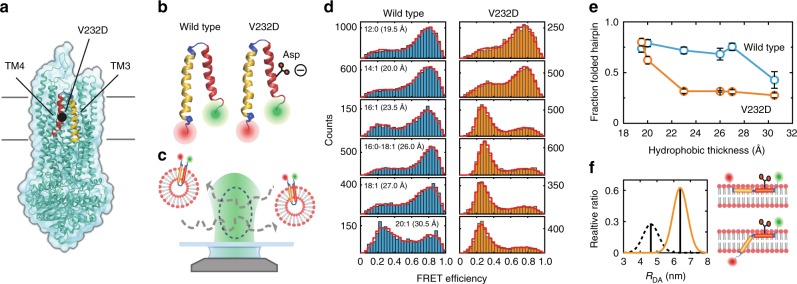
CFTR wild-type and V232D mutant TM3/4 hairpin folding probed by single-molecule FRET. **a** Structure of CFTR^3^ highlighting the position of the V232D mutation in TM3/4 (yellow/red). **b** Schematic representation of the wild-type (left) and V232D (right) TM3/4 helical-hairpin motifs comprising CFTR’s transmembrane helices TM3 (yellow) and TM4 (red). **c** Schematic of the single-molecule FRET approach for investigating hairpin conformations. Shown are single fluorescently labeled TM3/4 hairpin molecules reconstituted into phospholipid vesicles freely diffusing through the observation volume of the confocal microscope. **d** FRET efficiency histograms of wild-type (blue) and V232D TM3/4 (orange) in PC lipid vesicles with 12:0, 14:1, 16:1, 16:0–18:1 (POPC), 18:1, and 20:1 acyl chains. Distances between the acyl chain C-2 atoms are indicated as measures of hydrophobic thicknesses^48^. PDA fits to the histograms are shown as red cityscapes. **e** Fraction of folded hairpin as function of hydrophobic thickness for wild-type TM3/4 (blue) and V232D TM3/4 (orange) as determined by PDA fits. Errors are standard deviations of the PDA chi-square minimization algorithm calculated from ten iterations. **f** Closed-state (black dashed) and open-state (orange solid) interfluorophore distance (*R*_DA_) distributions for V232D TM3/4 in POPC determined using PDA (left panel), in accordance with a fully extended interfacially bound hairpin or a partially inserted hairpin with TM3 being inserted and TM4 positioned atop the bilayer (right panels)

In addition to a deletion of residue F508 in the first nucleotide-binding domain, the transmembrane domains of CFTR represent a particularly vulnerable hotspot and frequent target of pathogenic misfolding mutations, especially when polar residues are introduced at positions within the hydrophobic lipid bilayer core. More than 300 point mutations in CFTR’s transmembrane helices have been described^4^, a majority of which promote aberrant protein folding. The cystic-fibrosis-phenotypic mutation V232D, which affects the center of CFTR’s fourth transmembrane helix (TM4) (Fig. 1a), belongs to a large group of disease-linked nonpolar-to-polar mutations within transmembrane domains that severely inhibit maturation of CFTR^8^. However, the molecular details of V232D-induced misfolding remain unclear, and conflicting models regarding the mechanism exist. Earlier, Therien et al.^9^ postulated that the formation of a non-native H-bond between the mutant’s carboxylate group of D232 in TM4 to the native carboxamide in Q207 of the third transmembrane helix (TM3) could abolish the conformational freedom of CFTR required for correct folding and channel dynamics—an inference based on the identical migration rates of the V232D hairpin and a corresponding disulfide-crosslinked wild-type hairpin in sodium dodecyl sulfate-polyacrylamide gel electrophoresis^10^. However, more recent studies indicate that the enhanced migration rate of V232D vs. wild type may be due to the “escape” of the polar TM4 Asp locus to the micelle surface^10^. In support of the latter interpretation, Loo and Clarke^11^ have suggested that misfolding of the V232D variant results from the disruption of native packing interactions, thereby trapping the protein in a misfolded state. Interestingly, they further showed that the folding defect caused by the V232D mutation can be rescued with the aid of small-molecule compounds, including the Food and Drug Administration-approved corrector drug Lumacaftor (also known as VX-809)^12^, to yield mature protein at the cell surface with native-like activity^11^. Yet the mechanism of action of these pharmacological correctors on mutant CFTR remains elusive; more generally, it is unclear whether they directly target the mutant protein to stabilize the native conformation or whether they exert their effects indirectly.

Here we exploit a helical-hairpin construct derived from full-length CFTR as a minimalist in vitro system that provides structural and mechanistic insights into V232D-induced misfolding and its rescue by the small-molecule corrector Lumacaftor. Specifically, we studied CFTR folding within the context of the TM3/4 hairpin motif comprising CFTR’s third and fourth transmembrane helices and their intervening extracellular loop (Fig. 1b, human CFTR residues 194–241). This helix–loop–helix construct represents the smallest unit that can be inserted autonomously by the translocon, as membrane-protein topogenesis in the endoplasmic reticulum is based on the pairwise integration of transmembrane segments^13^, and can reproduce transmembrane helix–helix interactions in vitro according to the two-stage model of membrane-protein folding^14^ to behave as independent folding domains—even when excised from the parent protein^15,16^. Through comparison of a hairpin carrying the V232D mutation with the wild-type hairpin, this minimal folding unit furnishes molecular-level insights into mutation-related effects and drug action without relying on full-length CFTR, which largely evades detailed in vitro scrutiny because it is notoriously difficult to obtain in sufficient quantities and purities and is too large and complex to pinpoint the local structural effects of point mutations. To track the folding status of hairpins in lipid bilayer membranes, we devised a single-molecule Förster resonance energy transfer (FRET) approach that probes the end-to-end distances of hairpins reconstituted in phospholipid vesicles. We find that the wild-type hairpin is well folded, whereas the V232D hairpin assumes an open conformation in bilayer thicknesses mimicking the endoplasmic reticulum. Addition of Lumacaftor reverses the aberrant opening of the V232D mutant hairpin to restore a compact fold as in the wild type. The observed membrane escape of the V232D mutant and its reversal by the chemical corrector Lumacaftor complement cell-based mutational analyses of the full-length protein, thereby providing in vivo/in vitro correlates of CFTR misfolding and drug-rescue action mechanisms.

## Results

### A single-molecule FRET assay to study CFTR hairpin folding

To study misfolding and pharmacological correction in TM3/4 helical hairpins, we developed a single-molecule FRET^17,18^ assay that probes structural changes affecting helical packing with subnanometer precision^19^. To this end, we engineered wild-type and V232D TM3/4 variants labeled with donor and acceptor fluorophores at the N- and C-termini of the hairpins and reconstituted the hairpins into liposomes (see Methods and Supplementary Information). We then employed single-molecule fluorescence spectroscopy to probe hairpin compactness by measuring the FRET efficiency between the two dyes (Fig. 1c). The use of pulsed-interleaved excitation (PIE)^20^ in combination with time-correlated single photon counting (Supplementary Fig. 1) allowed for fluorescence-aided sorting^21^ (Supplementary Fig. 2) and thus the creation of FRET efficiency histograms with only FRET-active molecules present^22,23^ (see Supplementary Methods). Importantly, because the FRET efficiency is recorded on single molecules, the obtained histograms sensitively report on coexisting conformational hairpin states and their relative occupancies, thus enabling a direct readout of structural changes imposed on helical packing upon mutation and drug action.

### Effect of membrane thickness and V232D mutation on CFTR hairpin folding

In a first set of experiments, we probed hairpin conformations in lipid bilayers composed of phosphatidylcholines (PCs) of various acyl chain lengths in order to modulate the hydrophobic thickness of the membrane systematically from very thin (e.g., 12:0 PC) to very thick (e.g., 20:1 PC) bilayers, including values mimicking the endoplasmic reticulum membrane (e.g., 16:0–18:1 PC; i.e., 1-palmitoyl-2-oleoyl-*sn*-glycero-3-PC, POPC). TM3/4 hairpins adopted α-helical structures when reconstituted into POPC lipid bilayers and displayed typical signatures of membrane association, as shown by circular dichroism and Trp fluorescence spectroscopy (see Supplementary Figs. 3 and 4). Since TM3 contains both a surface-exposed as well as a membrane-embedded Trp residue (see Supplementary Fig. 5), the observed distance insensitivity of Trp quenching by dibrominated lipids along the bilayer normal (Supplementary Figs. 3c and 4a) strongly indicates a transmembrane position of this segment. This is supported by acrylamide quenching experiments, which confirm a membrane-embedded state of TM3 (Supplementary Fig. 4b). Together with the tight packing of the helices (see below and Supplementary Table 1), this in turn implies a membrane-inserted topology of the entire wild-type TM3/4 hairpin. This is further supported by free-energy calculations^24^ and segmental hydropathy analysis^25^ of wild-type TM3 and TM4 (Supplementary Table 2). Finally, the sensitivity of the hairpin equilibrium to bilayer width also strongly supports a membrane-inserted state (see below and Fig. 1d).

FRET efficiency histograms of the wild-type hairpin under all conditions exhibited bimodal distributions with a major high-FRET population and a minor low-FRET population (Fig. 1d, blue). The high-FRET efficiency population is centered at ~0.8 (Supplementary Table 1), which translates into a mean inter-dye distance of ~4.4 nm consistent with a tightly packed, well-folded hairpin. The wild-type hairpin was only moderately sensitive to changes in membrane thickness, and only very thick membranes led to partial opening, indicating that the equilibrium toward the open, low-FRET state shifts only if the strain imposed by hydrophobic mismatch becomes high. Thus, across a broad range of membrane thicknesses, the wild-type hairpin exists in an equilibrium between a compact, folded structure and an open-state conformation, with the equilibrium lying on the side of the compact conformation determined by tight helix–helix interactions.

By contrast, the V232D mutation (Fig. 1d, orange) assumed a closed, high-FRET hairpin conformation only in very thin membranes, as increasing bilayer thickness drastically inverted the equilibrium toward the open state. To exclude the possibility that the observed changes in FRET efficiency populations arise from acceptor-quenched subpopulations due to labeling heterogeneity, we performed correlative analyses of the relative donor fluorescence lifetime (*τ*_D(A)_/*τ*_D(0)_) vs. FRET efficiency (Supplementary Fig. 6). This showed that the two populations observed in the histograms did not originate from labeling heterogeneity caused by acceptor quenching but stemmed from two conformations of the wild-type and V232D hairpins that change their relative occupancies in response to bilayer thickness and upon mutation.

Quantification of open-state and closed-state fractions using probability-distribution analysis (PDA) (Fig. 1d, red cityscapes) showed that the folded state in the V232D hairpin is ~50% less populated than the wild-type hairpin across a wide range of membrane thicknesses (Fig. 1e). Accordingly, hairpin stability in POPC as reflected in the Gibbs free-energy change of hairpin opening, is favorable in wild-type TM3/4 (Δ*G*°_wild type_ = −1.9 kJ/mol) but turns positive upon mutation (Δ*G*°_V232D_ = +1.9 kJ/mol). The interfluorophore distance in the open state amounts to ~6.3 nm (Fig. 1f, Supplementary Table 1). This distance is consistent with a scenario where both helices of the V232D mutant lie embedded atop the bilayer or one where TM3 is inserted diagonally in the membrane while TM4 lies atop the bilayer. The latter scenario is deduced from the much stronger quenching behavior of TM3’s two Trp residues by dibrominated lipids in V232D TM3/4 as compared with the wild-type hairpin in addition to a slight distance dependence of the quenching behavior (see Supplementary Figs. 3c, 4a). Application of the aqueous membrane-impermeable quencher acrylamide also suggests a membrane-embedded state of TM3 in the mutant hairpin with both Trp residues residing in a well-shielded, protective environment within the bilayer (see Supplementary Fig. 4b). Together, these observations suggest a tilted transmembrane orientation of TM3 likely as a consequence of an interfacial positioning of V232D TM4. Support for an interfacial location of V232D TM4 comes from free-energy calculations^24^. Wild-type TM4 has an interface-to-octanol transfer free energy of Δ*G*_I→O_ = –5.3 kJ/mol; therefore, bilayer insertion into a TM position is thermodynamically favorable. By contrast, Δ*G*_I→O_ = +7.0 kJ/mol for V232D TM4, thus lending credence to an interfacial position, which remains favorable over the aqueous state by Δ*G*_W→I_ = –16.1 kJ/mol. Moreover, segmental hydropathy analyses of wild-type vs. V232D TM4 suggest a decreased preference for membrane insertion of the mutant segment (Supplementary Table 2). Additionally, the hydrophobic moment of TM4 rises from 0.40 in the wild type to 3.34 in the V232D mutant, which facilitates the adoption of an interfacial location of the mutant.

Taken together, the V232D mutation strongly destabilizes the compact hairpin state that is expected to mimic the native conformation in full-length CFTR. Our observation of facilitated hairpin opening in V232D TM3/4 therefore speaks against an earlier proposed scenario that involves a non-native H-bond lock between TM3 and TM4^9^. Presumably, the free energy released upon formation of a putative H-bond in V232D TM3/4 is not sufficient to offset the free-energy penalty incurred by inserting the mutant’s D232 carboxylate group into the hydrocarbon core of the membrane. Rather, membrane escape of the V232D hairpin, as observed in our experiments, is in line with recent cell-based mutational analyses of full-length CFTR, which proposed that the loss of nonpolar interactions among several transmembrane helices is responsible for misfolding^11^. Hence, our minimalist in vitro system is able to reproduce misfolding effects observed in vivo, thereby providing molecular insights into the structural consequences imposed by the V232D mutation.

### Lumacaftor restores a compact fold of the V232D mutant

**Fig. 2:**
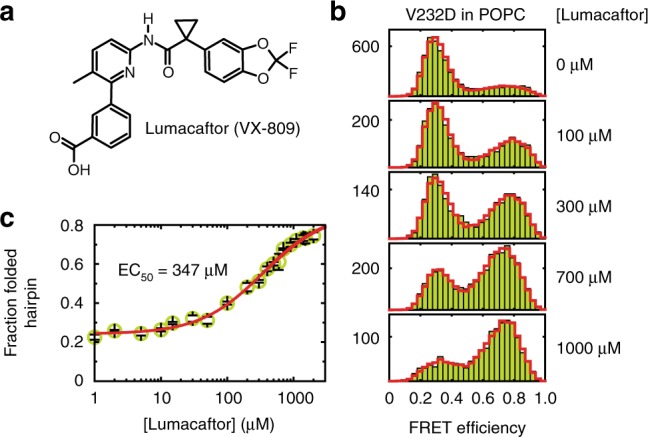
Reversal of V232D TM3/4 hairpin opening by Lumacaftor. **a** Structure of the pharmacological corrector Lumacaftor (VX-809). **b** FRET efficiency histograms of V232D TM3/4 (chartreuse) in POPC vesicles at increasing concentrations of VX-809. PDA fits are shown as red cityscapes. **c** Dose–response curve of VX-809 action on V232D TM3/4 (chartreuse). Depicted is the fraction of compact hairpin conformation as a function of corrector concentration. The apparent affinity was determined by a sigmoid fit (red), which yielded an EC_50_ of ~350 µM. Other fit parameters were *f*_F,min_ = 0.24, *f*_F,max_ = 0.87, and *n* = 0.91. Errors in **c** are standard deviations of the PDA chi-square minimization algorithm calculated from ten iterations

On this premise, we explored whether the helical-hairpin motif also captures the rescuing activity of a small-molecule corrector to provide deeper insights into its mechanism of action. In vivo, the folding defect caused by the V232D mutation is restored by the corrector Lumacaftor (Fig. 2a), which enhances the delivery of matured protein to the cell surface^11,26^. The promiscuity of Lumacaftor for correcting various cystic fibrosis-causing mutants, with high selectivity reported for the CFTR protein, implies that there may be multiple mechanisms or binding sites at play. To date, various groups have narrowed down the interaction of Lumacaftor to the first nucleotide-binding domain and/or the first transmembrane domain of CFTR^11,26–28^. Possible drug-action mechanisms could either be that Lumacaftor acts on CFTR’s transmembrane domains already during co-translational folding, thereby preventing misfolding, or that it acts on misfolded CFTR posttranslationally, thereby restoring a functional topology.

To test Lumacaftor action in vitro, we reconstituted the V232D hairpin into vesicles composed of POPC. In the absence of the corrector, the mutant hairpin predominantly adopted an open conformation (Fig. 2b), but titration with Lumacaftor reversed hairpin opening to restore a compact state with FRET levels close to those of the wild-type hairpin. We obtained a typical dose–response dependency with an EC_50_ value (i.e., the drug concentration producing 50% of the maximum effect) of ~350 µM (Fig. 2c), from which we derived an apparent free-energy change accompanying Lumacaftor addition similar to wild-type levels (Δ*G*°_V232D,Lumacaftor_ ≈ −2.5 kJ/mol at saturation levels). Hence, Lumacaftor can act directly on the helix–helix interaction affected by the V232D mutation, as it rescues the compact fold of the TM3/4 hairpin in an in vitro system in the absence of cellular translocon and sorting machineries. This is supported by the observation that Lumacaftor also exerted a slight stabilizing effect on the wild-type hairpin (Supplementary Fig. 7), substantiating the notion that the chemical corrector aids in establishing helix–helix interactions in the TM3/4 hairpin motif. Conversely, the non-cystic-fibrosis-related drug Azelastine, a selective histamine-H1 receptor antagonist used in the symptomatic treatment of allergic rhinitis, which has the same octanol/water partition coefficient as Lumacaftor (log*P* = 4.4), did not exert a rescuing activity on the mutant hairpin (Supplementary Fig. 8), indicating specificity of Lumacaftor action on the TM3/4 segment.

## Discussion

**Fig. 3:**
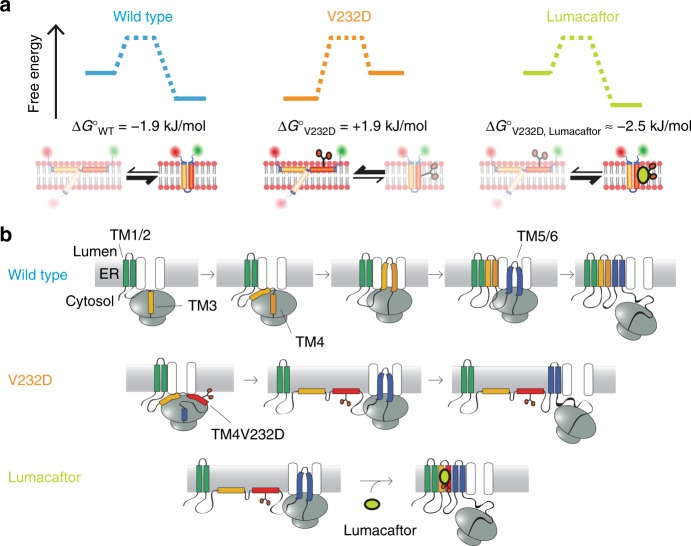
TM3/4 hairpin equilibria and mechanistic models for V232D-induced misfolding and drug rescue by Lumacaftor. **a** TM3/4 hairpin equilibria of wild type, V232D, and V232D upon rescue with Lumacaftor. Δ*G*° values are given for POPC bilayers. **b** Upper row: Model of wild-type CFTR topogenesis at the endoplasmic reticulum, adapted from Kim and Skach^14^. Transmembrane segments are integrated in a pairwise manner into the endoplasmic reticulum membrane. After integration of TM1/2, TM3 and TM4 simultaneously insert as a helical hairpin as TM3 encodes an inefficient signal sequence and thus cooperates with TM4 to translocate the intervening extracellular loop into the membrane. Middle row: Topogenesis model for misfolding of the V232D mutant. For clarity, one example of V232D TM3/4 positioning is depicted here, with both helices interfacially bound, with the alternative being a partially inserted state for TM3 (see **a** and Fig. 1f). The latter is not shown as it represents a highly unlikely situation that would necessitate an inverted topology of TM3/4 with the hydrophilic intervening loop between TM3 and TM4 spanning the hydrophobic core region of the membrane. Lower row: Model of reversal of V232D misfolding by small-molecule corrector Lumacaftor (see Discussion for potential mechanisms)

A summary of the results obtained on TM3/4 hairpin conformation and stability in POPC, whose thickness reflects that of the endoplasmic reticulum, is presented in Fig. 3a. While the wild-type hairpin mainly adopts a compact, membrane-inserted conformation consistent with tight helix–helix interactions—thereby validating the hairpin as a model for tertiary folding—, incorporation of an Asp residue in mid-TM4 shifts the equilibrium toward the open state in the V232D mutant, which can be reversed by the addition of Lumacaftor to restore a compact hairpin state.

From our results on the V232D mutation of CFTR, we propose—based on current models of full-length CFTR biogenesis^13^ (Fig. 3b, upper row)—a mechanistic model of V232D CF pathogenesis in which the free-energy penalty due to an anionic residue in TM4 impairs membrane insertion of the TM3/4 helical hairpin, thereby disrupting the native topology of the channel protein (Fig. 3b, middle row). This scenario is supported by the finding that TM3 and TM4 in wild-type CFTR are inserted into the membrane simultaneously in the form of a helical hairpin, as the TM3 sequence comprises only an inefficient translocation signal for the extracellular loop that connects TM3 and TM4^13,29^. The exchange of a hydrophobic for a charged Asp residue in the center of TM4 further drastically decreases the hydrophobicity and increases the amphiphilicity of this helical segment, thereby impairing membrane insertion of the hairpin and likely leaving TM3 and TM4 in an interfacial position at the cytoplasmic side of the endoplasmic reticulum membrane. This model of conformational misfolding is in accord with the observation that the hydrophobic pocket among transmembrane segments 4 (TM4), 5 (TM5), and 6 (TM6) is not formed in the V232D mutant^11^, resulting in an altered topology that inhibits CFTR maturation and traps the protein in a partially folded, intermediate state at the endoplasmic reticulum^11^. While the TM3/4 hairpin system is a useful minimalist model of protein tertiary contacts to rationalize mutation-induced effects on CFTR misfolding, we note that the in vivo topogenesis of CFTR is far more complex than represented by self-insertion of isolated helical-hairpin segments. For example, insertion of TM1/2 precedes insertion of TM3/4 into the membrane^13^ (Fig. 3b, upper row). Thus, in the early stages of in vivo folding, transmembrane helical segments 3 and 4 may form helical contacts with transmembrane helices 1 and 2 (TM1/2). This, in turn, may influence the negative impact of the V232D mutation on misfolding, for example, by enabling the D232 locus in mutant CFTR to favorably interact with neighboring, positively charged residues during biogenesis to stabilize, at least partially, a topically correct fold. Such a scenario could explain the mild form of cystic fibrosis caused by the V232D mutation^30^, in which maturation is not completely abolished but levels at ~20% of normal CFTR maturation^11,31^.

Addition of the small-molecule corrector Lumacaftor reverses misfolding of the V232D mutant and enables interfacially misfolded TM3/4 to regain a compact state thereby facilitating proper folding of the full-length protein (Fig. 3b, lower row). This finding is consistent with the concept that small-molecule correctors modulate the conformation of a region of CFTR to enhance global protein folding and assembly^6^. Thus the rescuing activity of Lumacaftor (and, possibly, other pharmacological chaperones) can be attributed to a “direct” effect enabled by their preferential stabilization of the native or near-native state of CFTR, as opposed to “indirect” effects mediated by quality-control or protein-sorting mechanisms. That large-scale topological changes can take place even after membrane insertion has been confirmed for other multispanning membrane proteins. For example, the membrane topology of LacY is modulated by changes in lipid composition, which trigger transmembrane segment flipping and thus topology inversion even after insertion of LacY into the membrane^32^. Moreover, as the assembly of a transmembrane domain influences the folding of adjacent cytosolic domains and vice versa, the action of Lumacaftor on transmembrane domains might also contribute to the rescue and restabilization of the common ΔF508 CFTR mutant^13,33^.

A question that arises from these findings is which mechanisms are at play in allowing the lipid-intolerant Asp locus in TM4—in the presence of the corrector—to partition into the membrane to restore a compact fold. Lumacaftor has several polar sites, including a benzoic acid carboxylate substituent and an amide bond, and has been shown to partition and stably reside within the lipid bilayer^34^. While our results do not pinpoint the protein–corrector binding site, Lumacaftor might be able to shield the negative impact of the polar mutation through polar interactions of its hydrophilic groups with the Asp carboxylate group, particularly in a core membranous region where p*K*_a_ values become significantly shifted^35^. Alternatively, Lumacaftor may bind elsewhere to TM4, thereby raising the overall hydrophobicity of the segment to overcome the unfavorable insertion of the Asp residue, likely promoting interhelical side chain–side chain interactions, including H-bonding interactions with the Q207 carboxamide in TM3^9^. A third scenario could be that Lumacaftor exerts its effect by interacting with the lipid bilayer membrane. Lumacaftor has been shown to homogeneously distribute throughout phospholipid membranes, thereby inducing structural perturbations of the bilayer profile and affecting membrane stability^34^. In this way, the changed physicochemical properties of the membrane may favor integration of the polar TM4 segment to restore a compact fold, similar to what has been observed by thinning bilayer thickness through modulating acyl chain length (Fig. 1d).

In conclusion, we have shown that a minimal hairpin motif, essentially the simplest model of protein tertiary contacts, provides mechanistic insights into the structural and energetic effects of a disease-causing CFTR mutation and its pharmacological rescue without relying on the full-length protein, thereby affording in vivo/in vitro correlates for understanding CFTR misfolding and drug rescue across scales. The present approach should be applicable to other CFTR mutants and chemical correctors, as the mechanism of pairwise transmembrane segment insertion also applies to transmembrane helices TM1/2, TM5/6, TM9/10, and TM11/12 of CFTR^13^. Together with the large library of cystic-fibrosis-phenotypic mutations in synthetic CFTR helical hairpins at hand^10,36^, this constitutes a promising platform for the quantitative analysis of CFTR transmembrane domain misfolding and the impact of drug-rescue effects. The most clinically useful small molecules bind directly to mutant CFTR; hence, our minimalist in vitro system is ideally suited for identifying such compounds because it eliminates chaperones and other cellular components involved in CFTR biogenesis as potential targets. Moreover, our approach might pave the way for the design of in vitro assays to test mechanistic hypotheses on other disease-related helical membrane proteins, whose misfolding can be restored with the aid of pharmacological chaperones and where a better understanding of in vivo/in vitro correlations is desirable to facilitate the development of mechanism-based therapies.

## Methods

A list of reagents and chemicals including the full names of lipids (all obtained from Avanti Polar Lipids, Alabaster, AL, USA) are given in Supplementary Information.

### Preparation and reconstitution of fluorescently labeled TM3/4 hairpins

CFTR wild-type and V232D mutant TM3/4 hairpin variants for site-specific double labeling were constructed with two Cys residues placed at the N- and C-terminal ends of the CFTR sequence (see Supplementary Fig. 5). Hairpins were produced and purified as previously described^9,37,38^ with minor modifications as detailed in Supplementary Information (see also Supplementary Fig. 9). Labeling with FRET donor (ATTO532; Atto-Tec, Siegen, Germany) and acceptor (ATTO647N; Atto-Tec) fluorophores was performed following published procedures^39,40^ as described in Supplementary Information. Hairpins were reconstituted into large unilamellar vesicles (LUVs) (see also Supplementary Fig. 10 and Supplementary Table 3) to yield proteoliposomes with a protein-to-vesicle molar ratio of <1:10 (i.e., less than every tenth LUV contained one hairpin molecule). Details on hairpin design, production, purification, labeling, LUV preparation, and hairpin reconstitution are given in Supplementary Information.

### Single-molecule FRET measurements

Experiments were carried out using a single-molecule confocal fluorescence microscope as previously described^41,42^ and detailed in Supplementary Information. Measurements were performed at 24 °C in buffer (50 mM Tris, pH 7.4) on freely diffusing proteoliposomes at an effective hairpin concentration of <100 pM. For measurements with Lumacaftor, sample solutions were supplemented with corrector at concentrations ranging from 1 to 2000 µM. Samples were incubated for at least 12 h prior to measurements. Details on instrumentation and data analysis including single-molecule burst selection; data reduction using stoichiometry, ALEX–2CDE^43^, and asymmetric burst filtering^22^; calculation of FRET efficiencies; and analysis of FRET efficiency histograms by PDA are provided in Supplementary Information.

### Fitting of dose–response curve

The concentration-dependent effect of Lumacaftor on the mutant hairpin was analyzed in terms of a four-parameter logistic regression model according to

1 $$f_F{\mathrm{ = }}f_{{\mathrm{F,max}}} + \frac{{f_{{\mathrm{F,min}}}{\mathrm{-}}f_{{\mathrm{F,max}}}}}{{{\mathrm{1 + }}\left( {\frac{{\left[ {{\mathrm{Lumacaftor}}} \right]}}{{{\mathrm{EC}}_{{\mathrm{50}}}}}} \right)^n}}$$

where *f*_F_ is the fraction of folded hairpin at a given Lumacaftor concentration [Lumacaftor], *f*_F,min_, and *f*_F,max_ are the folded fractions at, respectively, baseline (i.e., zero concentration) and maximum response (i.e., at infinite concentration) levels, EC_50_ is the concentration where the response is half-maximal, and *n* is an arbitrary “shape” parameter describing the slope of the dose–response curve. Best-fit parameter values {*f*_F,min_, *f*_F,max_, EC_50_, *n*} were estimated by nonlinear least-squares fitting in Matlab (Mathworks, Natick, MA, USA).

### Calculation of transfer free energies, hydrophobic moments, and segmental hydrophobicities

Transfer free energies were calculated using Membrane Protein Explorer (MPEx, http://blanco.biomol.uci.edu/mpex/)^24^, a hydropathy analysis software based on experimentally determined whole-residue hydrophobicity scales^44^ that allows for a thermodynamic dissection of membrane-association and -insertion energetics of transmembrane segments from sequence information. We used the “MPEx Totalizer” tool to determine the water-to-octanol (Δ*G*_W→O_), water-to-interface (Δ*G*_W→I_), and interface-to-octanol (Δ*G*_I→O_) transfer free energies of wild-type and V232D TM4. Calculations were based on the membrane-embedded segments of the wild-type (i.e., SAFAGLGFLIVLALFQAGL) and the V232D mutant (i.e., SAFAGLGFLIDLALFQAGL) comprising residues 222–240 of CFTR as predicted from earlier studies^37^. Additionally, we employed the “MPEx Totalizer” module to determine the hydrophobic moment of wild-type and V232D TM4 as a measure of the amphiphilicity of the α-helices^45^. Average hydrophobicities of wild-type and V232D TM3 and TM4 segments were calculated based on the Liu–Deber hydrophobicity scale using the “TM finder” tool^25^.

### Circular dichroism and Trp fluorescence spectroscopy

The secondary structure of unlabeled wild-type and V232D mutant hairpins reconstituted in POPC LUVs was analyzed by far-ultraviolet circular dichroism spectroscopy. Membrane association of hairpins was probed by Trp fluorescence spectroscopy. Trp quenching experiments using dibrominated POPC analogs and Stern−Volmer analysis were performed as previously described^46,47^ and detailed in Supplementary Information. The solvent accessibility of Trp residues was probed by acrylamide quenching experiments and Stern–Volmer analysis. A molar protein-to-lipid ratio of 1:500 was used in all experiments. Measurements were performed at room temperature in 50 mM sodium phosphate, pH 7.4. Experimental details on circular dichroism and Trp fluorescence spectroscopy as well as Trp fluorescence quenching experiments using dibrominated lipids and acrylamide are described in Supplementary Information.

### Code availability

All code is available from the authors upon request.

## Electronic supplementary material

Supplementary Information Final

## Data Availability

All relevant data that are not in the article or Supplementary Information are available from the authors upon request.
