# Supplementary material for: A minimal helical-hairpin motif provides molecular-level insights into misfolding and pharmacological rescue of CFTR
Source: Commun Biol. 2018 Sep 28;1:154. doi: 10.1038/s42003-018-0153-0 (PMC6162264; doi:10.1038/s42003-018-0153-0)
Supplement: Supplementary file 1 — Supplementary Information Final [file 42003_2018_153_MOESM1_ESM.pdf]

SUPPLEMENTARY INFORMATION

A minimal helical-hairpin motif provides molecular-level insights into misfolding and pharmacological rescue of CFTR

Georg Krainer[1,2,§,*], Antoine Treff[1,§], Andreas Hartmann[1], Tracy A. Stone[3,4], Mathias Schenkel[1], Sandro Keller[2], Charles M. Deber[3,4,*], Michael Schlierf[1,*]

[1] B CUBE—Center for Molecular Bioengineering, Technische Universität Dresden, Arnoldstr. 18, 01307 Dresden, Germany.

[2] Molecular Biophysics, Technische Universität Kaiserslautern (TUK), Erwin-Schrödinger-Str. 13, 67663 Kaiserslautern, Germany.

[3] Division of Molecular Medicine, Research Institute, Hospital for Sick Children, 686 Bay Street, Toronto, ON, M5G 0A4, Canada.

[4] Department of Biochemistry, University of Toronto, 1 King's College Circle, Toronto, ON, M5S 1A8, Canada.

* Correspondence to:

Georg Krainer: georg.krainer@tu-dresden.de

Charles M. Deber: deber@sickkids.ca

Michael Schlierf: michael.schlierf@tu-dresden.de

[§] These authors contributed equally to this work.

Supplementary Figures

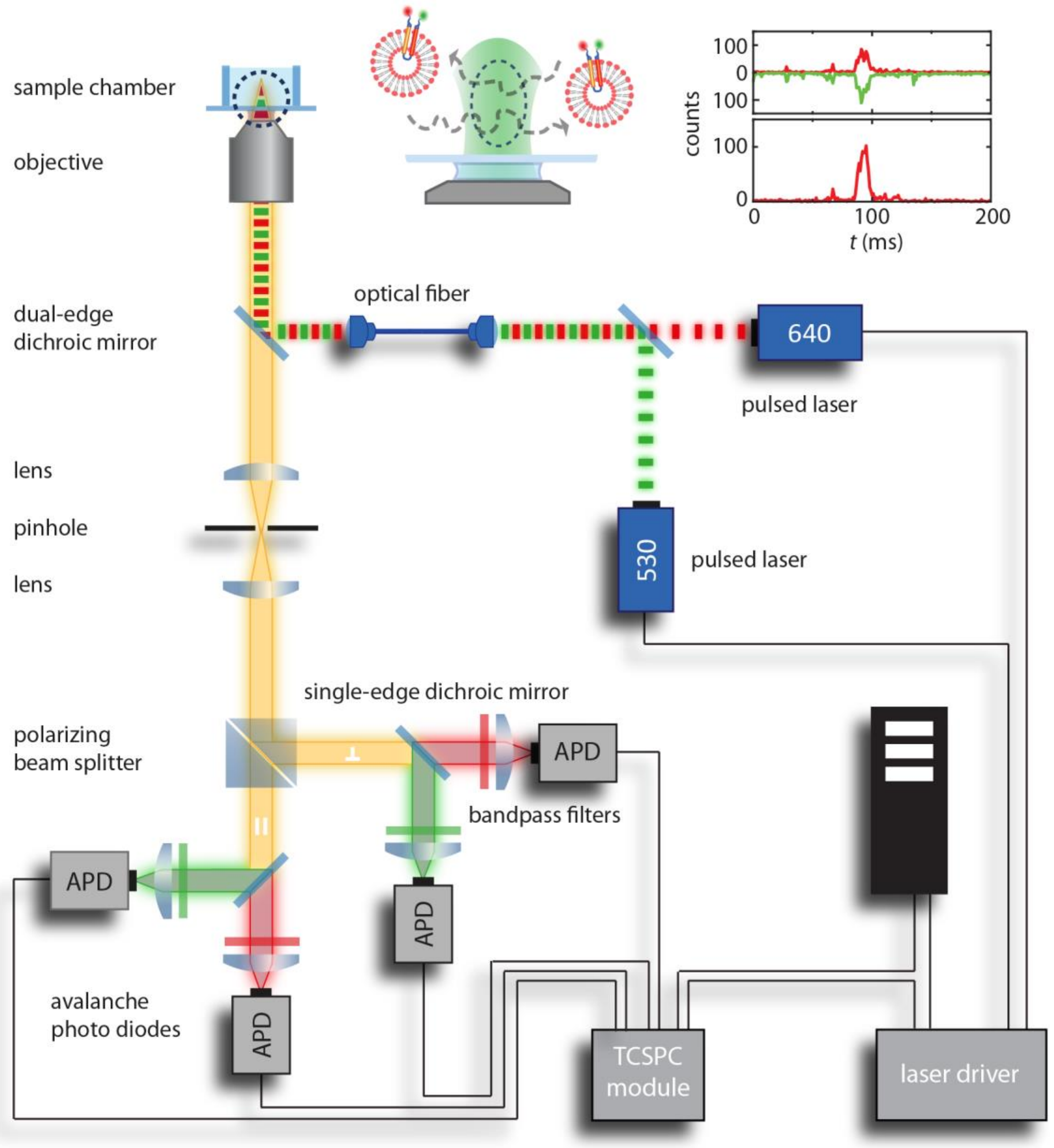

Supplementary Figure 1. Schematic of the confocal fluorescence spectroscopy setup equipped with pulsed interleaved excitation (PIE) and time-correlated single-photon counting (TCSPC). Left inset: fluorescently labeled hairpin molecules reconstituted into lipid vesicles freely diffusing through the confocal volume, resulting in emission of photons. Right inset: representative fluorescence intensity time trace (1-ms binning) with donor and acceptor emission after donor excitation (top, green and red) and acceptor emission after acceptor excitation (lower, red).

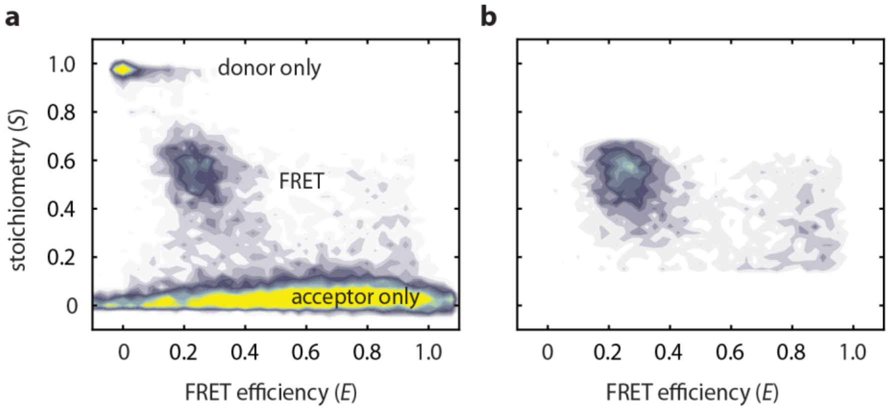

Supplementary Figure 2. Two-dimensional stoichiometry (*S*) versus FRET efficiency (*E*) plot of V232D TM3/4 in POPC (a) before and (b) after applying stoichiometry, ALEX–2CDE, and asymmetric burst filtering. Donor/acceptor-labeled FRET molecules are located at $S \approx 0.5$, while donor-only and acceptor-only molecules can be found at $S \approx 1$ and $S \approx 0$, respectively. Applying $0.15 < S < 0.7$, ALEX-2CDE < 7 and $|T_X^{Dexc} - T_A^{Aexc}| < 50$ µs removes donor- and acceptor-only subpopulations as well as blinking and photobleaching events.

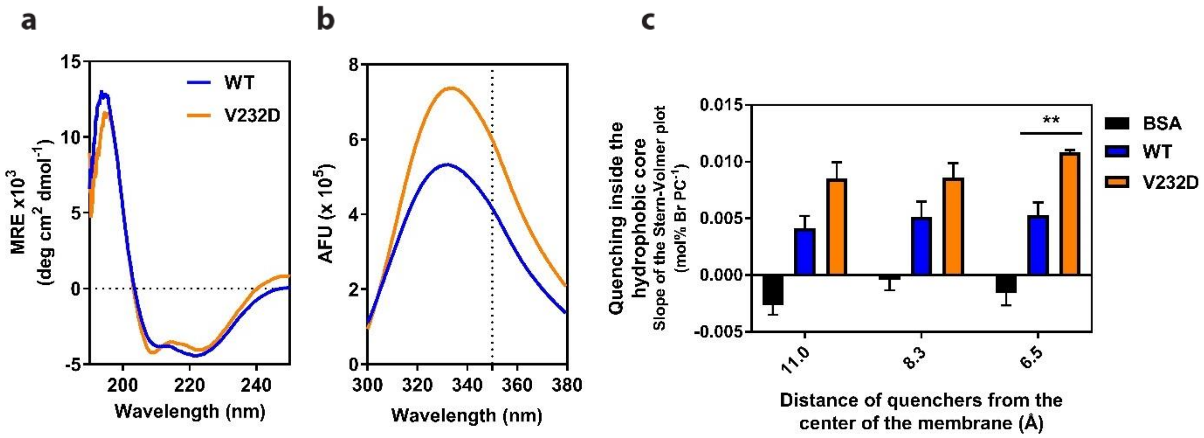

Supplementary Figure 3. Circular dichroism and Trp fluorescence of wild-type (WT) and mutant V232D hairpins. (**a**) Circular dichroism spectra of WT (blue) and mutant V232D (orange) TM3/4 hairpins (10 µM protein, 5 mM lipid). Both hairpins adopted α-helical structures when reconstituted into POPC lipid bilayers. (**b**) Trp fluorescence spectra of WT (blue) and mutant V232D (orange) TM3/4 hairpins (10 µM protein, 5 mM lipid). WT TM3/4 displays an average Trp emission maximum of 332 nm, with the mutant V232D displaying a slightly more red-shifted Trp emission maximum of 334 nm. Samples were excited at 280 nm, and emission was recorded at 300–380 nm. Trp emission maximum in water is depicted as a dashed vertical line at 350 nm. (**c**) Trp burial within the membrane was recorded by plotting the slopes of Stern−Volmer plots derived from fluorescence quenching as a function of quencher content in the membrane (see Supplementary Fig. 4a) vs. distance of quencher from the center of the bilayer (Å). Both WT (blue) and mutant V232D (orange) TM3/4 hairpins show significant association with the lipid bilayer at all membrane depths, implying that both constructs are at least partially inserted into the membrane. Because TM3 contains both a surface-exposed and a membrane-embedded Trp residue (Supplementary Fig. 5), the distance-independent quenching behavior of the WT hairpin along the bilayer normal indicates a transmembrane position of TM3. The more but significantly distance-dependent quenching behavior of the V232D hairpin as compared with the WT hairpin indicates that TM3 in V232D TM3/4 is inserted not in a perfectly transmembrane but slightly offset position. The stronger quenching behavior observed for V232D TM3/4 as compared with the WT hairpin (in particular at 6.5 Å from the center of the lipid bilayer) indicates that the two Trp residues are more accessible to the bromine moieties and, additionally, are buried more deeply in the bilayer than in WT TM3/4. This can be reconciled by a hairpin-opening scenario in which TM4 leaves the membrane and causes TM3, as a consequence of the interfacial positioning of TM4, to assume a more diagonal transmembrane position in the membrane, which is more accessible to the lipid quenchers because protective helix–helix interactions are abolished. BSA (black) was used as a negative control for membrane association. Error bars represent standard errors of the mean (s.e.m.). Significant differences between WT and mutant are depicted with ** ($p < 0.01$).

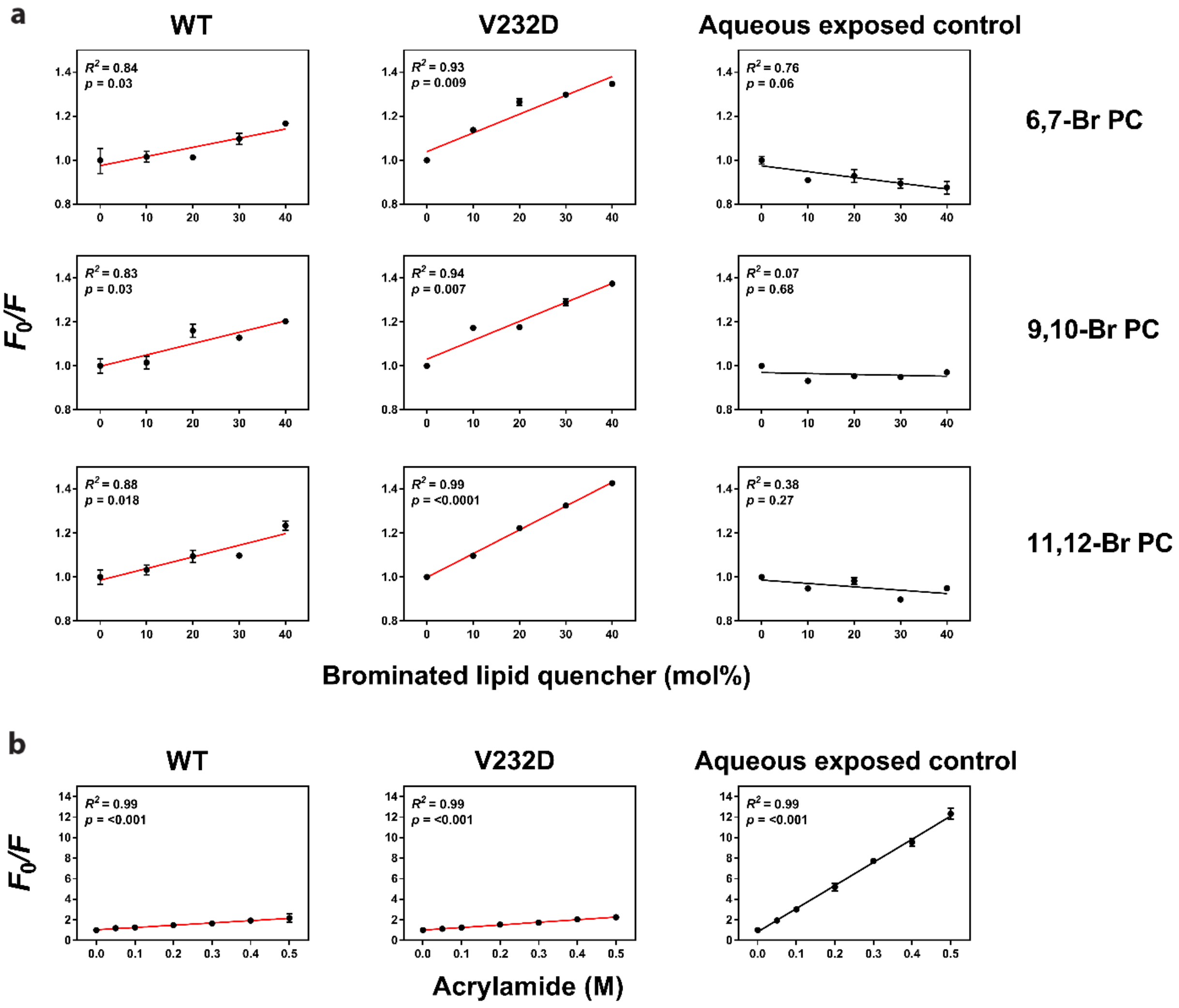

Supplementary Figure 4. Stern−Volmer plots of Trp fluorescence quenching in wild-type (WT) and V232D helical hairpins with dibrominated lipids and in the presence of acrylamide. (**a**) Trp fluorescence intensity for WT TM3/4 (left), V232D TM3/4 (center), and BSA (right, negative control for insertion) was measured at increasing concentrations of quencher (0, 10, 20, 30, 40 mol% Br PC). Quenching was reported as F_0/F, where F_0 represents fluorescence with no quencher and F represents fluorescence in the presence of quencher. Trend lines labeled in red indicate regression analyses. Coefficients of correlation (R^2) and corresponding p-values are given in each graph. Error bars represent propagated errors. Data represent averages of 3 independent experiments. (**b**) Trp fluorescence intensity for WT (left) and V232D (center) TM3/4 in POPC lipid bilayers was measured at increasing concentrations of acrylamide (0.05–0.5 M). A water-exposed peptide (right) in a lipid-free environment is included for relative comparison to the degree of acrylamide-Trp quenching of the reconstituted hairpins (see Methods). Quenching was reported as described in (a). Stern–Volmer constants (slope of the linear fit), which provide information on the degree of quenching, are as follows: WT, 2.24 ± 0.072; V232D, 2.53 ± 0.05; aqueous control, 22.5 ± 0.45. No departure from linearity was detected for the linear regressions depicted above. These results indicate that the two Trp residues in the WT and the mutant V232D hairpin reside in similar membrane-embedded environments and are highly protected by the membrane from the aqueous quencher, as indicated by the relatively small Stern–Volmer constants. Data shown represents the average of 3 independent experiments, error is reported as standard deviation (s.d.), $p < 0.001$ for all 3 linear regressions.

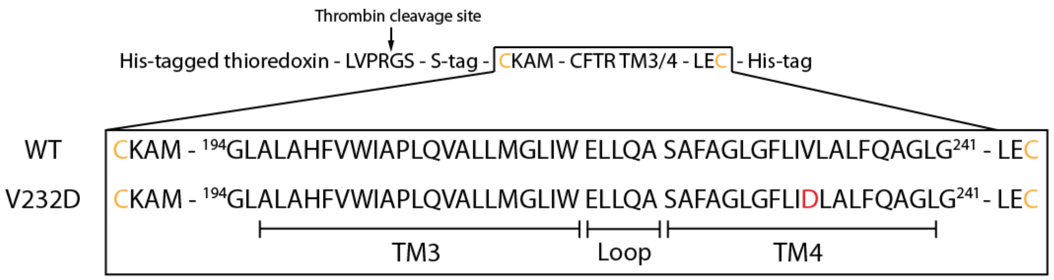

Supplementary Figure 5. Design and sequence of CFTR wild-type (WT) and V232D hairpin fusion constructs. The V232D mutation in TM4 is indicated in red, and the two Cys residues used for labeling are highlighted in orange.

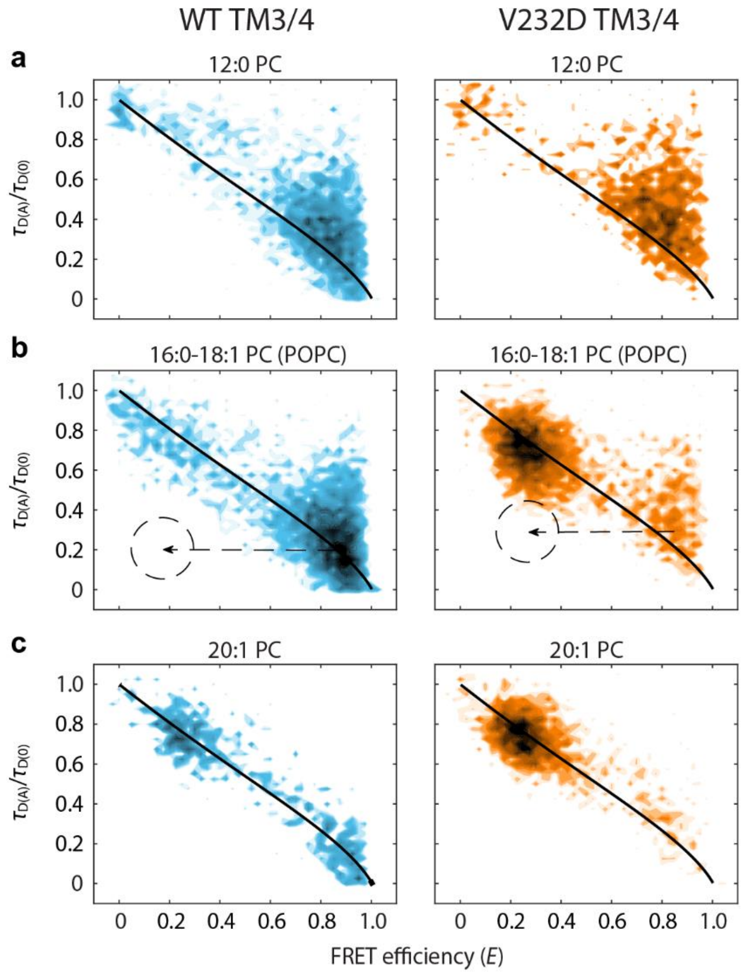

Supplementary Figure 6. Two-dimensional plot of relative donor fluorescence lifetime ($\tau_{D(A)}/\tau_{D(0)}$) versus FRET efficiency (*E*) of wild-type (WT) (blue) and V232D (orange) hairpins in (a) 12:0 PC, (b) 16:0–18:1 PC (POPC), and (c) 20:1 PC. Open and closed hairpin populations are located along the 'static FRET line'[1,2] (black line) indicating that FRET populations do not originate from unwanted labeling heterogeneity caused by acceptor quenching (dashed area) but from two conformations of the hairpins that change in response to bilayer thickness and upon mutation.

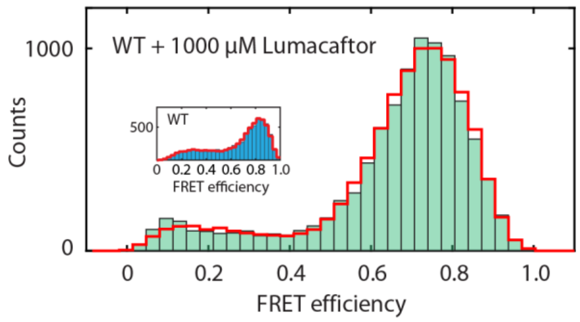

Supplementary Figure 7. FRET efficiency histogram of wild-type (WT) TM3/4 in POPC and in the presence of 1000 µM Lumacaftor (turquoise). A PDA fit is shown as red cityscape. The fraction of folded hairpin amounts to ~0.86 as compared with ~0.68 for the WT hairpin in the absence of Lumacaftor (cf. inset, blue; see also Supplementary Table 1), indicating that Lumacaftor exerts a stabilization effect on the WT hairpin.

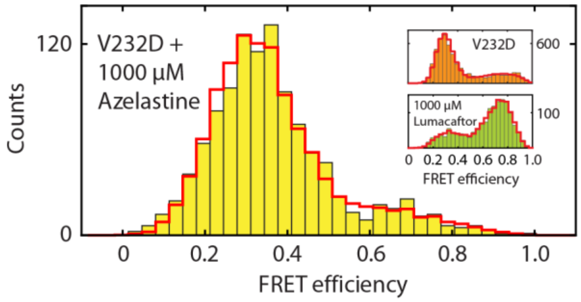

Supplementary Figure 8. FRET efficiency histogram of V232D TM3/4 in POPC and in the presence of 1000 µM Azelastine (yellow). A PDA fit is shown as red cityscape. The fraction of folded hairpin amounts to ~0.17, which is similar to the state occupancy of the V232D hairpin in the absence of Lumacaftor (cf. inset, orange; see also Fig. 1f). Thus, Azelastine does not exert any rescuing activity on the V232D hairpin as observed for 1000 µM Lumacaftor (cf. inset, chartreuse; see also Fig. 2b).

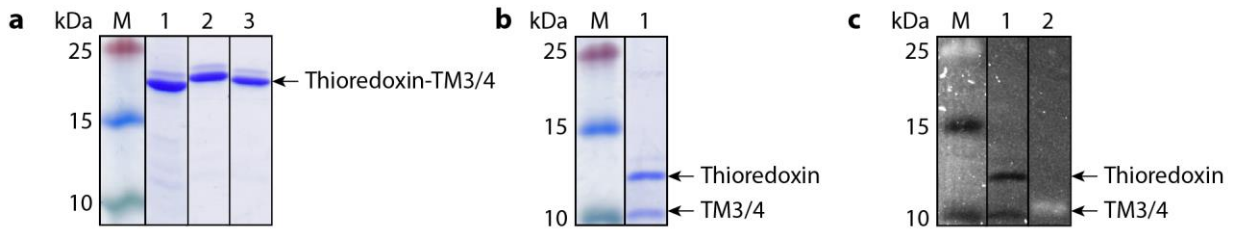

Supplementary Figure 9. Purification and labeling of the wild-type (WT) TM3/4 hairpin followed by SDS-PAGE. (**a**) Thioredoxin–TM3/4 fusion protein (23.4 kDa) after purification by IMAC (lane 1), after heat treatment and centrifugation (lane 2), and after buffer exchange (i.e., before thrombin digestion) (lane 3). (**b**) Cleaved thioredoxin–TM3/4 fusion protein (thioredoxin, 13.9 kDa and WT TM3/4, 9.5 kDa) after thrombin digestion. (**c**) In-gel fluorescence analysis of cleaved thioredoxin–TM3/4 fusion protein before (lane 1) and after labeling (lane 2) with ATTO532 and ATTO647N and consecutive purification by SEC. Fluorescence visualization (white bands) was achieved using a ChemiDoc MP Imaging System (Bio-Rad, Hercules, CA, USA) with green epi-illumination and a 605/50 emission filter; non-florescent bands appear black. M, molecular-weight protein marker (PageRuler Plus Prestained Protein Ladder, Thermo Fisher); the molecular weights are indicated (kDa). Samples were analyzed on 16% SDS-PAGE gels and stained with Coomassie Brilliant Blue.

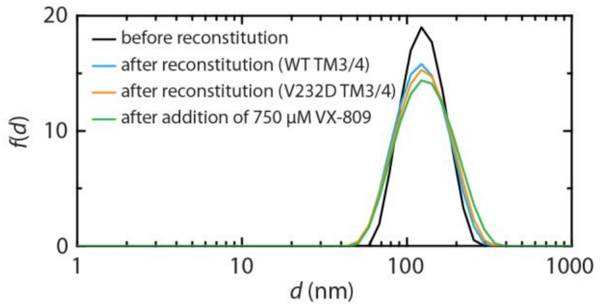

Supplementary Figure 10. DLS of POPC LUVs before and after reconstitution with wild-type (WT) or V232D mutant hairpins and in presence of Lumacaftor. Intensity-weighted particle size distribution functions (*f*(*d*)) versus hydrodynamic diameter (*d*) of POPC LUVs before reconstitution (black), after reconstitution with WT TM3/4 (blue) or V232D TM3/4 (orange), and after addition of 750 µM Lumacaftor.

Supplementary Tables

Supplementary Table 1. Extracted parameters of wild-type (WT) and V232D TM3/4 hairpins in PC LUVs obtained from two-state PDA histogram fits[a]

Lipid	TM3/4	E^*_F	E^*_O	R_F (nm)	R_O (nm)	σ^*_F	σ^*_O	f_F
**12:0 PC**	WT	0.765±0.007	0.323±0.019	4.52±0.03	6.23±0.09	0.49±0.02	0.75±0.11	0.74±0.04
	V232D	0.719±0.008	0.317±0.028	4.70±0.03	6.25±0.13	0.50±0.02	0.50±0.07	0.80±0.04
**14:1 PC**	WT	0.754±0.009	0.279±0.027	4.56±0.04	6.45±0.14	0.57±0.02	0.78±0.09	0.79±0.03
	V232D	0.739±0.008	0.352±0.017	4.62±0.03	6.09±0.07	0.45±0.02	0.68±0.07	0.62±0.04
**16:0 PC**	WT	0.744±0.008	0.258±0.013	4.60±0.03	6.56±0.08	0.53±0.02	0.63±0.10	0.72±0.03
	V232D	0.732±0.010	0.283±0.004	4.65±0.04	6.42±0.02	0.53±0.04	0.37±0.03	0.32±0.02
**16:0–18:1 PC**	WT	0.800±0.012	0.353±0.044	4.37±0.05	6.09±0.20	0.45±0.05	0.76±0.16	0.68±0.06
	V232D	0.713±0.013	0.298±0.002	4.73±0.05	6.34±0.01	0.63±0.02	0.28±0.01	0.32±0.01
**18:1 PC**	WT	0.760±0.006	0.273±0.022	4.54±0.03	6.48±0.12	0.59±0.02	0.62±0.10	0.75±0.04
	V232D	0.725±0.014	0.272±0.003	4.68±0.05	6.48±0.02	0.57±0.04	0.41±0.02	0.31±0.02
**20:1 PC**	WT	0.769±0.040	0.304±0.023	4.49±0.16	6.32±0.12	0.60±0.09	0.62±0.11	0.43±0.08
	V232D	0.710±0.016	0.267±0.004	4.74±0.06	6.51±0.02	0.60±0.04	0.43±0.02	0.28±0.02

[a] E^*_F and E^*_O are the apparent FRET efficiencies of the folded and open states, R_F and R_O are the average inter-fluorophore distances of the folded and open states, σ^*_F and σ^*_O are the widths of the folded and open states, and f_F is the fraction of the folded state. Errors are standard deviations of the chi-square minimization algorithm calculated from 10 iterations.

Supplementary Table 2. Transfer free energies[a] and average hydrophobicities[b] of wild-type (WT) and V232D TM3 and TM4

TM segment	$\Delta G_{W\rightarrow I}$ (kJ/mol)	$\Delta G_{W\rightarrow O}$ (kJ/mol)	$\Delta G_{I\rightarrow O}$ (kJ/mol)	Φ_{LD}
**WT TM3**	–23.7	–40.1	–16.4	2.0
**WT TM4**	–21.0	–26.3	–5.3	1.7
**V232D TM4**	–16.1	–9.1	+7.0	1.4

[a] $\Delta G_{W\rightarrow I}$, $\Delta G_{W\rightarrow O}$, $\Delta G_{I\rightarrow O}$ denote the water-to-interface, water-to-octanol, and the interface-to-octanol transfer free energies of WT and V232D TM4, respectively, as determined using the 'MPEx Totalizer' tool[3]. Calculations were based on the membrane-embedded segments of WT (i.e., SAFAGLGFLIVLALFQAGL) and V232D (i.e., SAFAGLGFLIDLALFQAGL) comprising residues 222–240 of CFTR as predicted from earlier studies[4].

[b] Φ_{LD} denote hydrophobicity values determined using the Liu–Deber hydrophobicity scale using the 'TM Finder' tool[5].

Supplementary Table 3. Size of PC LUVs before and after reconstruction with wild-type (WT) and V232D TM3/4 as measured by DLS

Lipid	TM3/4	Average diameter (nm)[a]	PDI[b]
**12:0 PC**			
**before reconstitution**		118.0	0.037
**after reconstitution**	WT	122.0	0.160
	V232D	125.7	0.154
**14:1 PC**			
**before reconstitution**		119.6	0.068
**after reconstitution**	WT	119.5	0.078
	V232D	120.6	0.106
**16:0 PC**			
**before reconstitution**		118.5	0.067
**after reconstitution**	WT	118.2	0.115
	V232D	115.2	0.085
**16:0–18:1 PC**			
**before reconstitution**		121.1	0.058
**after reconstitution**	WT	115.0	0.103
	V232D	116.4	0.108
**18:1 PC**			
**before reconstitution**		116.3	0.126
**after reconstitution**	WT	114.4	0.081
	V232D	114.7	0.060
**20:1 PC**			
**before reconstitution**		124.9	0.070
**after reconstitution**	WT	134.1	0.204
	V232D	124.0	0.076

[a] z-average hydrodynamic diameter

[b] PDI, polydispersity index

Supplementary Methods

Reagents and chemicals

All chemicals were purchased in the highest purity available. The phospholipids 1,2-dilauroyl-*sn*-glycero-3-phosphocholine (12:0 PC), 1,2-dimyristoleoyl-*sn*-glycero-3-phosphocholine (14:1 (Δ9-Cis) PC), 1,2-dipalmitoleoyl-*sn*-glycero-3-phosphocholine (16:1 (Δ9-Cis) PC), 1-palmitoyl-2-oleoyl-*sn*-glycero-3-phosphocholine (16:0–18:1 PC, POPC), 1,2-dioleoyl-*sn*-glycero-3-phosphocholine (18:1 (Δ9-Cis) PC), 1,2-dieicosenoyl-*sn*-glycero-3-phosphocholine (20:1 (Δ11-Cis) PC) were obtained in powder form from Avanti Polar Lipids (Alabaster, AL, USA). Dibrominated phospholipids 1-palmitoyl-2-(6,7-dibromo)stearoyl-*sn*-glycero-3-phosphocholine (6,7-Br PC), 1-palmitoyl-2-(9,10-dibromo)stearoyl-*sn*-glycero-3-phosphocholine (9,10-Br PC), and 1-palmitoyl-2-(11,12-dibromo)stearoyl-*sn*-glycero-3-phosphocholine (11,12-Br PC) were obtained in chloroform dissolved aliquots from Avanti Polar Lipids. Lumacaftor (VX-809) was obtained from Selleck Chemicals LLC (Houston, TX, USA) with a purity of 99.07% (Batch No. S156502). ATTO532 and ATTO647N maleimide were from Atto-Tec (Siegen, Germany). Ampicillin, biotin, $CaCl_2$, dithiothreitol (DTT), glucose, imidazole, isopropyl-*β*-D-1-thiogalactopyranoside (IPTG), lysogeny broth (LB), $MgSO_4$, thiamin, NaCl, tris(hydroxymethyl)aminomethane (Tris), and tris(2-carboxyethyl)phosphine (TCEP) were from Carl Roth (Karlsruhe, Germany). Azelastine hydrochloride (Ph. Eur. Reference Standard, Order No. Y0000326), dimethyl sulfoxide (DMSO), lauryldimethylamine *N*-oxide (LDAO), M9 minimal salt, *β*-mercaptoethanol (*β*-ME), phosphate-buffered saline (PBS), 2,2,2-trifluoroethanol (TFE) and Triton X-100 were from Sigma–Aldrich (St. Louis, MO, USA). Benzonase, bovine serum albumin (BSA), chloroform, methanol, and thrombin were from Merck (Darmstadt, Germany).

Preparation of fluorescently labeled TM3/4 hairpins

Design. CFTR wild-type and V232D mutant TM3/4 hairpins were produced as thrombin-cleavable His-tagged thioredoxin–TM3/4 fusion proteins encompassing residues 194−241 of human CFTR, which corresponds to CFTR's third and fourth transmembrane helices and the intervening extracellular loop (Supplementary Fig. 5)[4,6]. Two Cys residues were placed at the N- and C-terminal ends of the CFTR sequence for conjugation to thiol-reactive fluorophores, and the native Cys residue at position 225 was replaced by Ala to prevent unspecific labeling. In the V232D mutant hairpin, the native Val residue at position 232 was exchanged to an Asp residue. Hairpins produced from these constructs have N-terminal and C-terminal extensions inherent to the pET32a vector that encode an S-tag epitope and a His-tag sequence, respectively, leaving final constructs of 88 residues after thrombin cleavage.

Production. Wild-type and V232D fusion proteins were recombinantly produced from previously described pET32a expression vectors (Merck, Darmstadt, Germany) following published procedures with minor modifications[4,6]. Briefly, the plasmids carrying an

ampicillin resistance marker were transformed into *Escherichia coli* BL21 (DE3) cells (Life Technologies, Darmstadt, Germany), and starter cultures were inoculated from single colonies, grown over night at 37°C in LB medium (containing 100 µg/mL ampicillin), and then used for inoculation of expression cultures in M9 medium minimal medium (supplemented with 100 µg/mL ampicillin). M9 minimal medium was prepared from M9 minimal salt supplemented with 1 mM $MgSO_4$, 0.1 mM $CaCl_2$, 0.001 % (*w*/*v*) thiamine, 0.001 % (*w*/*v*) biotin, and 3 g/L glucose. Cell cultures were grown to an OD_{600} of ~0.7, then induced by the addition of IPTG to a final concentration of 0.1 mM, and cultivated for a further 16 h at 16°C. Cells were harvested by centrifugation, washed with PBS, and after centrifugation either directly used for protein extraction or stored at −80°C.

Purification. The extraction and purification of hairpin constructs were adapted from published procedures[4,6]. The cell pellet was thawed on ice and resuspended in breakage buffer (0.1% (*w*/*v*) Triton X-100, 20 mM Tris, 150 mM NaCl, 5 mM imidazole, pH 8.0), which was freshly supplemented with 0.1 µL/mL Benzonase and 10 mM *β*-ME. Resuspended cells were lysed using an Emulsiflex high-pressure homogenizer (Avestin, Ottawa, ON, Canada), followed by centrifugation for 15 min at 17,600 *g* and 4°C to remove cell debris. For purification via immobilized-metal-ion affinity chromatography (IMAC), the soluble fraction of the cell lysate was applied to a Ni-NTA resin (Qiagen, Hilden, Germany) that had been pre-equilibrated with binding buffer (0.1% (*w*/*v*) Triton X-100, 20 mM Tris, 150 mM NaCl, 5 mM imidazole, pH 8.0). After incubation for 1.5 h at room temperature under gentle rocking, the resin was applied to gravity flow columns and, after collection of the flow-through, was washed extensively with washing buffer (0.1% (*w*/*v*) Triton X-100, 20 mM Tris, 300 mM NaCl, pH 8.0) containing 5, 15, 25, and 50 mM imidazole, followed by an additional wash step with binding buffer containing 50 mM imidazole. His-tagged proteins were eluted with elution buffer (0.1% (*w*/*v*) Triton X-100, 20 mM Tris, 150 mM NaCl, pH 8.0) containing 400 mM imidazole. Purification was monitored by sodium dodecyl sulfate polyacrylamide gel electrophoresis (SDS-PAGE) (Supplementary Fig. 9a, lane 1), and the protein concentration was assessed by comparison of band intensities using a BSA standard of known concentration, yielding a concentration of ~1 µM. Eluted fractions were pooled, and the sample was further purified by heat treatment for 10 min at 70°C, which resulted in the precipitation of remaining contaminants while the heat-resistant thioredoxin–TM3/4 fusion proteins remained in solution[7]. Heat-treated samples were centrifuged at 18,400 *g* for 10 min at 4˚C, and the supernatant was collected (Supplementary Fig. 9a, lane 2). For proteolytic removal of the thioredoxin solubility tag, the buffer was first exchanged into cleavage buffer (0.1% (*w*/*v*) Triton X-100, 50 mM Tris, 150 mM NaCl, 1.5 mM TCEP, pH 8.0) using PD10 desalting columns (GE Healthcare, Chicago, IL, USA) (Supplementary Fig. 9a, lane 3) before the sample was incubated with thrombin (1.6 units per milliliter of sample) at 4°C for at least 48 h under gentle rocking. Cleavage was confirmed by SDS-PAGE (Supplementary Fig. 9b, lane 1).

Labeling. For labeling of hairpin constructs with FRET donor (ATTO532) and acceptor (ATTO647N) fluorophores, the sample solution after thrombin cleavage was mixed with a ~20-fold molar excess of the corresponding maleimide-functionalized dye. The pH of the labeling reaction was adjusted to 7.25 prior to labeling to yield buffer conditions of 0.1% (*w*/*v*) Triton X-100, 50 mM Tris, 150 mM NaCl, 1.5 mM TCEP, pH 7.25. After incubation for 3 h at room temperature, the reaction was quenched with 5 mM DTT, and unreacted fluorophores, cleaved thioredoxin, as well as uncleaved protein were separated from the cleaved and dye-labeled wild-type and V232D TM3/4 constructs with the aid of size-exclusion chromatography on a Superdex 75 10/300 GL (GE Healthcare) in buffer containing 0.1% (*w*/*v*) Triton X-100, 50 mM Tris, 150 mM NaCl, 1.5 mM TCEP, pH 8.0, yielding fluorecently labeled hairpins at final concentrations of ~1 µM (wild-type TM3/4) and ~0.33 µM (V232D TM3/4). Purity was confirmed by SDS-PAGE (Supplementary Fig. 9c, lanes 1 and 2).

Preparation of large unilamellar phospholipid vesicles

For the preparation of LUVs, lipid powders were weighed on a high-precision XP Delta Range microbalance (Mettler Toledo, Greifensee, Switzerland) and dissolved in a 2:1 (*v*/*v*) chloroform/methanol mixture at a concentration of 20 mg/mL. After mixing, the solvent was removed under a gentle nitrogen flow for 5 min, followed by vacuum drying for 2 h. Dried lipid cakes were suspended in buffer (50 mM Tris, pH 7.4), vortexed for 5 min, and incubated for 1 h under shaking to yield dispersions at a final lipid concentration of 20 mM. Hydrated lipid suspensions were subjected to 10 freeze/thaw cycles by alternately placing the sample into liquid nitrogen and a 42°C water bath, followed by 31-fold extrusion through polycarbonate filters with a pore diameter of 100 nm using a Mini-Extruder (Avanti Polar Lipids) according to the manufacturer's protocol. Formation of LUVs was confirmed by dynamic light scattering (DLS) (Malvern Zetasizer Nano S, Malvern, UK), yielding LUVs with a unimodal vesicle size distribution around a mean hydrodynamic diameter of ~120 nm (Supplementary Table 3, Supplementary Fig. 10).

Reconstitution of TM3/4 hairpins into phospholipid vesicles

Reconstitution was performed at room temperature by first diluting fluorescently labeled hairpins ~80-fold (wild-type TM3/4) or ~30-fold (V232D TM3/4) into buffer containing 10 mM LDAO, 50 mM Tris, pH 7.4, resulting in residual Triton X-100 concentrations far below its critical micellar concentration ($CMC_{Triton\ X\text{-}100}$ = 0.22 mM, i.e. ~0.012% (*w*/*v*)). The LDAO-solubilized fluorescently labeled hairpins were then added under gentle agitation to an equal volume of 20 mM (lipid concentration) freshly prepared LUVs, resulting in 10 mM (lipid concentration) lipid vesicles containing residual LDAO at a subsolubilizing concentration (i.e., 5 mM) and a final protein concentration of 5–7 nM. This translates into a protein-to-vesicle molar ratio of <1:10 (i.e., less than every 10th LUV contains one hairpin molecule). The reconstitution mixture was incubated for 10 min at 50°C, followed by incubation at room temperature for 1.5 h during which the sample was diluted 5-fold with buffer (50 mM Tris, pH 7.4) in three steps to reach a final LDAO concentration far below

its CMC (CMC_{LDAO} = 1.5 mM) and to also further dilute Triton X-100. To further reduce detergent concentrations, samples were dialyzed against a 1000-fold excess volume of buffer (50 mM Tris, pH 7.4) using a Slide-A-Lyzer Mini dialysis device (Thermo Fisher, Waltham, MA, USA) with a molar-mass cutoff of 3.5 kg mol^{-1} for a total of 36 h during which the dialysate was exchanged three times. After dialysis, the proteoliposomes retained their narrow, unimodal size distribution profiles as confirmed by DLS (Supplementary Table 3, Supplementary Fig. 10).

Single-molecule FRET experiments

Instrumentation. Observations of single-molecule fluorescence were made on a custom-built dual-color and dual-polarization confocal setup based on an inverted microscope (Eclipse Ti-E, Nikon, Tokyo, Japan) (Supplementary Fig. 1). Donor and acceptor fluorophores were excited with linearly polarized 530-nm and 640-nm picosecond pulsed laser sources (LDH-P-FA-530L and LDH-D-C-640, both from PicoQuant, Berlin, Germany) driven in PIE mode at a total repetition rate of 50 MHz. The laser beams were coupled to a polarization-maintaining single-mode optical fiber (P3-488PM-FC-2, Thorlabs, NJ, USA), collimated (60FC-T-4-RGBV42-47, Schäfter und Kirchhoff, Hamburg, Germany), and focused by a water immersion objective (CFI Plan Apo WI 60x, NA 1.2, Nikon). Emitted fluorescent light was collected by the same objective, separated from the excitation light by a dual-edge dichroic mirror (zt532/642rpc, Chroma, Bellows Falls, VT, USA), and focused on a 50-µm pinhole (Thorlabs). Donor and acceptor photons were spectrally separated by single-edge dichroic mirrors (FF650-Di01, Semrock, USA) after a polarizing beam splitter (CM1-PBS251, Thorlabs), band-pass-filtered (FF01-582/75, Semrock, Rochester, NY, USA; ET700/75M, Chroma), and focused onto four single-photon-counting avalanche diodes (τ-SPADs, PicoQuant). Photons were registered by four individual TCSPC modules (Hydra Harp, PicoQuant) with a time resolution of 16 ps. Synchronization with the lasers for alternating excitation was accomplished with the aid of a diode laser driver (PDL828, PicoQuant).

Experimental conditions. Measurements were performed at 24°C in custom-built sample chambers on freely diffusing proteoliposomes by placing the confocal volume into solution at an axial position ~70 µm above the surface of the cover slide. The illumination power was 110 µW for excitation of the donor dye and 90 µW for direct excitation of the acceptor dye. Proteoliposomes were diluted approximately 20-fold in buffer (50 mM Tris, pH 7.4) to yield an effective hairpin concentration of <100 pM, which together with the slow diffusion time of the vesicles and an occupancy of less than one fluorescently labeled hairpin molecule per ten lipid vesicles ensured single-molecule conditions at appropriate burst rates. For experiments involving Lumacaftor, a stock solution of 100 mM Lumacaftor in DMSO was prepared, from which micromolar stock solutions of Lumacaftor in aqueous buffer (50 mM Tris, pH 7.4) were made. POPC proteoliposomes were diluted with the aqueous Lumacaftor stock solutions in buffer (50 mM Tris, pH 7.4) to yield an effective hairpin concentration of <100 pM at the desired corrector concentration ranging from 1 µM

to 2000 µM. Samples were incubated for at least 12 h prior to single-molecule FRET measurements. Proteoliposomes in the presence of Lumacaftor retained their narrow, unimodal size distribution profiles as confirmed by DLS (Supplementary Fig. 10). Experiments of wild-type and V232D hairpin in the presence of Lumacaftor (1000 µM) or Azelastine (1000 µM), respectively, were performed as described for the V232D mutant hairpin in the presence of Lumacaftor.

Data analysis. Analysis of single-molecule FRET experiments was performed with custom-written Matlab scripts (Mathworks, Natick, MA, USA). Single-molecule events were identified from the acquired photon stream as fluorescence bursts with a maximum inter-photon time of 30 µs containing a minimum total number of 100 photons after background correction and using a Lee filter of 4. To minimize the number of bursts stemming from fluorescence background (e.g., impurities) and multiple molecule events that display signals in the donor and acceptor channels, only bursts with a minimum number of 15 photons in the acceptor channel after donor excitation (D_{exc}) were retained. For measurements with Lumacaftor, filter settings were further adjusted to exclude bursts stemming from corrector autofluorescence in the donor channel. To this end, the minimum number of photons in the acceptor channel after acceptor excitation (A_{exc}) was set to 40 counts, and the total number of counts after D_{exc} had to exceed 50 photons to be retained as a significant burst event; additionally, the Lee filter was set to 10. After burst identification, the FRET efficiency (E) of each single-molecule burst was calculated according to $E = n_A/(n_D+n_A)$, where n_D and n_A are the numbers of photons detected in the donor and acceptor channels after D_{exc}, respectively. FRET efficiencies were corrected for background, direct acceptor excitation, channel cross-talk, as well as differences in detector efficiencies and quantum yields of the dyes as described previously[8]. Apparent FRET efficiencies (E^*) used in PDA analysis were calculated without applying the aforementioned corrections. Bursts were further analyzed for the stoichiometry ratio, $S = n^{Dexc}/(n^{Dexc}+n^{Aexc})$, where n^{Dexc} is the total number of counts after D_{exc} and n^{Aexc} is the total number of counts after A_{exc}. This allowed us to exclude molecules lacking an active donor or acceptor from further analysis by selecting only molecules showing an S value between 0.15 and 0.7. Additionally, an alternating laser excitation−two-channel kernel-based density distribution estimator (ALEX-2CDE)[9] was used to remove blinking and bleaching and further remove donor- and acceptor-only events; only bursts with an ALEX-2CDE score <7 were selected; for experiments with Lumacaftor the ALEX-2CDE score was set to <10. To further remove molecules affected by photobleaching, an asymmetric burst filter[2] was applied, which discards all bursts $|T_X^{Dexc}-T_A^{Aexc}| < 50$ µs, with T_A^{Aexc} and T_X^{Dexc} being the burst-averaged macroscopic photon arrival times in the acceptor channel after A_{exc} and in the donor and acceptor channel after D_{exc}, respectively. An exemplary two-dimensional S-E plot for V232D TM3/4 in POPC before and after applying stoichiometry, ALEX–2CDE, and asymmetric burst filtering is shown in Supplementary Figure 2. In measurements with Lumacaftor, a small acceptor subpopulation that was

quenched was filtered out by selecting only bursts with an acceptor lifetime of $2 < \tau_A < 10$ ns. From all remaining bursts, FRET efficiency histograms were constructed by projecting single-molecule events of the two-dimensional *S*-*E* distributions onto the FRET efficiency axis with bin widths of 0.033; for PDA, histograms with apparent FRET efficiencies were constructed as detailed below.

Analysis of FRET efficiency histograms with PDA. To quantify conformational equilibria from FRET efficiency histograms, we used PDA[10], a method that remodels experimentally obtained histograms with a theoretical distribution to retrieve state fractions as well as mean FRET efficiencies of underlying subpopulations. We employed a two-state static PDA to describe the equilibrium between the compact, folded (F) and the extended, open (O) hairpin conformations with state fractions f_F and $f_O = 1-f_F$ as well as mean FRET efficiencies E^*_F and E^*_O, respectively. The model also included Gaussian distance distributions for folded and open states to account for additional widths ($\sigma_{F,O}$) in excess of shot-noise broadening. A Monte Carlo (MC) sampling-based regression algorithm in Matlab was used to iteratively fit the experimental FRET efficiency histogram with the theoretical two-state static PDA histogram by chi-square minimization to optimize the fit parameters $\{f_F, \sigma_F, \sigma_O, E^*_F, E^*_O\}$. The experimental FRET efficiency histogram employed in PDA fitting was created by selecting single time-bin segments of equal duration (i.e., $T_b = 1.5$ ms) from the center of each fluorescent burst and calculating the apparent FRET efficiency for each bin. To construct the theoretical PDA FRET efficiency histogram from the fit parameters $\{f_F, \sigma_F, \sigma_O, E^*_F, E^*_O\}$, the following iterative four-step MC sampling procedure was carried out: First, a distribution of two FRET states representing the folded and open states was generated by drawing the number of molecules in each state (i.e., n_F and $n_O = N-n_F$, respectively) from the binomial distribution

$$P(n_F|f_F, N, K) = \binom{K\cdot N}{n_F} f_F^{\,n_F}(1-f_F)^{K\cdot N-n_F} \tag{S1}$$

considering the folded-state fraction f_F and the total number of molecules N derived from the experimental histogram multiplied by an oversampling factor of $K = 20$.

For each folded and open molecule, individual inter-dye distances ($R_{F,O}$) were drawn from a Gaussian distribution probability function

$$P(R_{F,O}) = \frac{1}{\sqrt{2\pi}\sigma_{F,O}} \exp\left(-\frac{\left(R_{F,O}-\langle R_{F,O}\rangle\right)^2}{2\sigma_{F,O}^{\ 2}}\right) \tag{S2}$$

with $\sigma_{F,O}$ describing the width of the distribution and $\langle R_{F,O}\rangle$ the average inter-fluorophore distance as translated from the mean FRET efficiencies E^*_F and E^*_O using $\langle R_{F,O}\rangle =$

$R_0 \sqrt[6]{\frac{1}{E^*_{F,O}} - 1}$. Here, R_0 is the Förster radius, which amounts to 5.5 nm for the Atto532/Atto647N dye pair used in our study. Individual distances obtained for each molecule were then back-translated into individual FRET efficiencies $\varepsilon_{F,O}$ according to

$$\varepsilon_{F,O} = \frac{1}{1 + \left(\frac{\langle R_{F,O} \rangle}{R_0}\right)^6}$$

For each molecule, the number of emitted acceptor photons $a_{F,O}$ was drawn from the binomial distribution

$$P(a_{F,O}|F,\, \varepsilon_{F,O}) = \binom{F}{a_{F,O}} \varepsilon_{F,U}{}^{a_{F,O}} \left(1 - \varepsilon_{F,O}\right)^{F - a_{F,O}} \qquad \text{(S3)}$$

considering the individual FRET efficiency $\varepsilon_{F,O}$ and the number of detected photons F for each molecule as derived from the experimental data. Finally, the theoretical PDA FRET efficiency histogram was collected from the ratios $a_{F,O}/F$ of all molecules in the folded and open states and averaged by K. The theoretical histogram was compared to the experimental histogram by assessing the goodness of the fit using the reduced chi-square value as calculated from the differences between experimental and theoretical histograms. This procedure was repeated by optimizing the free parameters $\{f_F,\, \sigma_F,\, \sigma_O,\, E^*_F,\, E^*_O\}$ until a minimum chi-square value was obtained.

Circular dichroism spectroscopy

To reconstitute hairpin samples for circular dichroism spectroscopy, freeze-dried powders of unlabeled wild-type and V232D TM3/4 constructs were first quantitated in TFE prior to addition of aqueous LDAO and subsequent lyophilization. The freeze–dried hairpin/LDAO powders were then brought up in buffer (50 mM sodium phosphate, pH 7.4) and added to freshly prepared LUVs following procedures as described above with minor alterations. Briefly, POPC was aliquoted from chloroform and dried into films prior to lyophilization, water washing, and reconstitution in phosphate buffer. The lipid suspension was freeze–thawed five times prior to extrusion through polycarbonate filters with a pore diameter of 200 nm. Extruded LUVs were left to equilibrate overnight before addition of LDAO-solubilized hairpins. Samples were heated to 50˚C for 10 min, then left at room temperature for 1.5 h. Samples were diluted with buffer (to decrease the final concentration of LDAO) before dialysis against a 1000-fold excess volume of buffer (50 mM sodium phosphate, pH 7.4) for a total of 36 h during which the dialysate was exchanged three times. Final protein and lipid concentrations were 10 µM and 5 mM, respectively, giving a protein-to-lipid ratio of 1:500. Circular dichroism spectra were recorded on a Jasco J-720 spectropolarimeter (Easton, MD, USA) at room temperature in the wavelength range 190–250 nm with a step size of 0.1 nm, a spectral bandwidth of 2 nm, a scanning sped of 50 nm/min, and a 1-mm path length quartz glass cuvette. Each sample was scanned 7 times and averaged. Spectra were blank corrected and represent

the average of 3 independently prepared samples. Raw ellipticity signal was converted to mean residue ellipticity (MRE) using standard formulae.

Trp fluorescence spectroscopy

Samples were prepared as for circular dichroism experiments at final hairpin concentrations of 10 μM and 5 mM lipid concentration, giving a protein-to-lipid molar ratio of 1:500. Experiments were performed at room temperature using a 10-mm path length quartz glass cuvette on a Photon Technology International fluorimeter (Kyoto, Japan). Samples were excited using an excitation wavelength of 280 nm. Emission spectra were recorded in the wavelength range of 300–400 nm with a step size of 2 nm. An excitation slit width of 2 nm and emission slit width of 4 nm was used. Each sample was scanned 7 times, and spectra were further processed by averaging, and blank subtraction. Each spectrum represents the average of 3 independently prepared samples.

For Trp quenching experiments using dibrominated POPC analogues, mixtures of 10, 20, 30, and 40 mol% 6,7-Br PC, 9,10-Br PC, or 11,12-Br PC in POPC were prepared as described above for pure POPC samples. Fluorescence quenching data were obtained on a SpectraMax i3x microplate spectrofluorimeter (Molecular Devices, Sunnyvale, CA, USA), reading from the top of 96-well opaque plates. Each well contained 250 μL of 5 μM protein, 2.5 mM total lipid (1:500 protein-to-lipid molar ratio). Trp residues were excited at 280 nm, and emission was scanned from 300 to 380 nm as an average of six accumulations with a 1 nm step size. The area under the full spectrum was used as a measure of intensity after subtracting a blank spectrum from a sample without protein. For Stern−Volmer analysis, the ratio of fluorescence intensity without quencher and with quencher (F_0/F) was plotted versus quencher concentration. The magnitude of the slope of a plot of these variables was taken as a measure of quenching by a given quencher[11]. All regression analyses were performed using GraphPad Prism, version 7.0 (GraphPad software, La Jolla, CA, USA). BSA was used as a negative control for insertion into POPC bilayers.

For acrylamide quenching experiments, hairpins were reconstituted into POPC lipid bilayers as described above. Each sample contained 10 μM hairpin and 5 mM lipid (1:500 protein-to-lipid molar ratio). Fluorescence quenching data were obtained on a PTI QuantaMaster 80 spectrofluorometer (Horiba, Kyoto, Japan) using a 10-mm pathlength quartz cuvette containing a stir-bar cage and micro-stir bar. Excitation slit widths were set to 2 nm and emission slit widths to 5 nm. Samples were excited at 295 nm and emission spectra were recorded between 310–360 nm as an average of 3 accumulations with a 1 nm step size at room temperature. The area under the full spectrum was used as a measure of intensity after subtracting a blank spectrum from a sample without protein. Analysis of Stern–Volmer slopes was carried out as described above. Acrylamide was added to stirring samples from a concentrated stock (5 M) and allowed to completely mix prior to recording a spectrum. A water-soluble peptide (sequence: KKK-

AASAAAALLWLLLAAASAA-KKK) dissolved in buffer alone (no lipid bilayers) was included for relative comparison of Stern–Volmer slopes.

Supplementary References

1. Sisamakis, E., Valeri, A., Kalinin, S., Rothwell, P. J. & Seidel, C. A. M. Accurate Single-Molecule FRET Studies Using Multiparameter Fluorescence Detection. *Methods Enzymol.* **475,** 455–514 (2010).
2. Kudryavtsev, V. *et al.* Combining MFD and PIE for Accurate Single-Pair Förster Resonance Energy Transfer Measurements. *ChemPhysChem* **13,** 1060–1078 (2012).
3. Snider, C., Jayasinghe, S., Hristova, K. & White, S. H. MPEx: a tool for exploring membrane proteins. *Protein Sci.* **18,** 2624–8 (2009).
4. Nadeau, V. G., Rath, A. & Deber, C. M. Sequence Hydropathy Dominates Membrane Protein Response to Detergent Solubilization. *Biochemistry* **51,** 6228–6237 (2012).
5. Deber, C. M. *et al.* TM Finder: a prediction program for transmembrane protein segments using a combination of hydrophobicity and nonpolar phase helicity scales. *Protein Sci.* **10,** 212–9 (2001).
6. Therien, A. G., Grant, F. E. & Deber, C. M. Interhelical hydrogen bonds in the CFTR membrane domain. *Nat. Struct. Biol.* **8,** 597–601 (2001).
7. McCoy, J. & La Ville, E. Expression and purification of thioredoxin fusion proteins. *Curr. Protoc. Protein Sci.* **Chapter 6,** Unit 6.7 (2001).
8. Hartmann, A., Krainer, G., Keller, S. & Schlierf, M. Quantification of Millisecond Protein-Folding Dynamics in Membrane-Mimetic Environments by Single-Molecule Förster Resonance Energy Transfer Spectroscopy. *Anal. Chem.* **87,** 11224–11232 (2015).
9. Tomov, T. E. *et al.* Disentangling Subpopulations in Single-Molecule FRET and ALEX Experiments with Photon Distribution Analysis. *Biophys. J.* **102,** 1163–1173 (2012).
10. Santoso, Y., Torella, J. P. & Kapanidis, A. N. Characterizing Single-Molecule FRET Dynamics with Probability Distribution Analysis. *ChemPhysChem* **11,** 2209–2219 (2010).
11. Bolen, E. J. & Holloway, P. W. Quenching of tryptophan fluorescence by brominated phospholipid. *Biochemistry* **29,** 9638–9643 (1990).
